# Dual-function g-C_3_N_4_ anchored Cu–ZnS hybrid nanostructures for sustainable energy storage and environmental remediation

**DOI:** 10.1039/d5ra03396a

**Published:** 2025-07-08

**Authors:** Eman A. Alabdullkarem, Junaid Khan

**Affiliations:** a Chemistry Department, College of Science, King Saud University Riyadh 11451 Saudi Arabia; b Department of Physics, Government Postgraduate College No. 1 Abbottabad Khyber Pakhtunkhwa Pakistan junaidkhan.nanotech@gmail.com; c Department of Higher Education Achieves and Libraries, Government of Khyber Pakhtunkhwa Peshawar Pakistan; d Department of Chemical and Biological Engineering, Gachon University 1342 Seongnam-daero Seongnam 13120 Republic of Korea

## Abstract

The mounting global imperative for sustainable energy storage and effective wastewater treatment necessitates the innovation of multifunctional materials capable of addressing both challenges in tandem. In the present work, we demonstrate the fabrication of a hybrid nanostructure comprising graphitic carbon nitride (g-C_3_N_4_) integrated with Cu–ZnS, strategically engineered for dual functionality in photocatalytic and supercapacitor domains. X-ray diffraction (XRD) analysis confirmed the successful formation of the Cu–ZnS/g-C_3_N_4_ composite, revealing a synergistic coexistence of hexagonal and cubic ZnS crystal phases. Morphological characterization illustrated a uniformly integrated architecture, wherein Cu–ZnS nanoparticles were homogeneously distributed across the g-C_3_N_4_ nanosheets. BET surface area analysis indicated a pronounced enhancement, reaching 148.16 m^2^ g^−1^, representing a 1.6-fold increase relative to pristine Cu–ZnS. The multifunctionality of the composite was substantiated through its superior performance in both energy storage and environmental remediation. Specifically, the optimized CuZnS-GCN25 electrode exhibited an impressive specific capacitance of 275 F g^−1^ at 1 A g^−1^, retained 92.5% of its capacitance over 10 000 charge–discharge cycles, and maintained 70% retention at an elevated current density of 20 A g^−1^ in a two-electrode configuration. In photocatalytic applications, CuZnS-GCN25 facilitated the efficient degradation of amoxicillin (AMX), achieving 92.4% removal under visible light within 60 minutes, consistent with pseudo-first-order kinetics (*k* = 0.029 min^−1^). These results highlight the significant potential of CuZnS-GCN25 as a high-efficiency, dual-purpose material for sustainable water treatment and advanced hybrid energy storage systems.

## Abstract 2

The mounting global imperative for sustainable energy storage and effective wastewater treatment necessitates the innovation of multifunctional materials capable of addressing both challenges in tandem.

## Graphical abstract

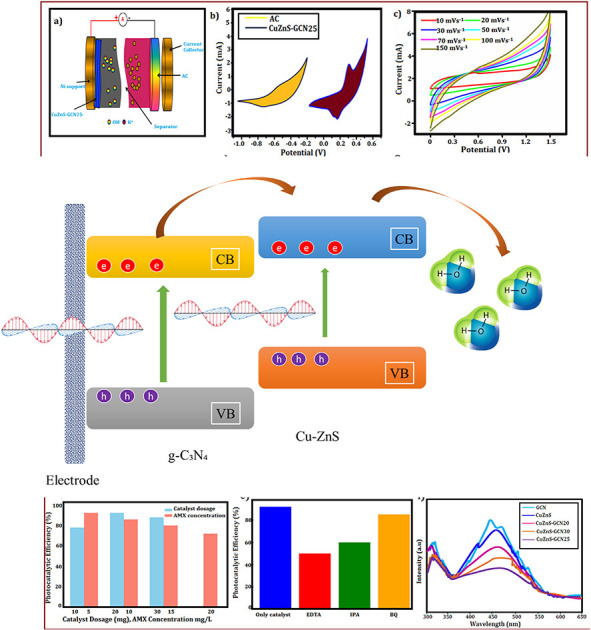

## 1. Introduction

Intensive industrialization, rapid urban expansion, and a growing global population have imposed considerable pressure on existing energy resources and the environment. In particular, the escalating energy demand, coupled with continued reliance on fossil fuels, has led to profound environmental consequences, including climate change and water contamination.^1,2^ The accelerating depletion of fossil fuel reserves has underscored the urgent need for efficient energy storage systems capable of supporting the integration of renewable energy sources. Concurrently, the discharge of hazardous organic pollutants—such as synthetic dyes, antibiotics, and heavy metals—from industrial and agricultural activities has led to severe water pollution, posing serious threats to both human health and ecological balance.^3,4^ Conventional energy storage technologies, including batteries, thermal energy storage, and compressed air energy storage, often suffer from limited efficiency, low power density, and environmental concerns arising from toxic components.^5^ Similarly, traditional wastewater treatment methods—such as adsorption, coagulation, filtration, and chemical precipitation—are frequently costly, energy-intensive, and inadequate for the removal of persistent organic pollutants.^6,7^ These pressing challenges highlight the necessity for multifunctional materials capable of simultaneously addressing energy storage and environmental remediation sustainably and efficiently.

Recent strides in nanotechnology have transformed the fields of energy storage and environmental remediation through the engineering of highly efficient nanoparticles with extensively tailored physicochemical properties.^8^ These innovations have paved the way for the development of more efficient, durable, and sustainable solutions to address pressing global energy and environmental challenges. Among emerging technologies, supercapacitors have garnered considerable attention as next-generation energy storage systems due to their high power density, rapid charge–discharge capability, and excellent cycle stability.^9^ Recent investigations have shown that the incorporation of conducting polymers, metal oxides (*e.g.*, MnO_2_, NiCo_2_O_4_), and carbon-based materials (*e.g.*, graphene, CNTs) has significantly enhanced their electrochemical performance.^10–12^ Consequently, these materials are increasingly suitable for integration into electric vehicles, portable electronics, and renewable energy infrastructure. Nevertheless, limitations such as low energy density and restricted long-term stability necessitate the design of hybrid nanostructures to achieve improved charge storage. In parallel, nanostructured photocatalysts have emerged as promising candidates for the degradation of organic pollutants and environmental purification under solar irradiation.^13^ Advancements in heterostructure-based photocatalysts have facilitated more effective charge separation and extended light absorption, thereby improving degradation efficiency.^14^ Despite these developments, challenges such as charge recombination in photocatalysts and limited energy density in supercapacitors persist, underscoring the critical need for multifunctional hybrid nanostructures capable of delivering synergistic performance across both domains.

Zinc sulfide (ZnS) has recently garnered significant attention as a semiconductor material due to its wide bandgap, excellent optical properties, and robust chemical stability, rendering it a promising candidate for energy storage and photocatalytic applications.^15^ However, the inherent limitations of pure ZnS—namely, its low electrical conductivity and rapid charge carrier recombination—restrict its efficacy in both photocatalytic and electrochemical domains. To overcome these constraints, the development of hybrid electrodes comprising transition metals and ZnS has emerged as a compelling approach.^16^ Such composite architectures can substantially enhance electrical conductivity, charge storage capacity, and cycling stability, thereby outperforming conventional single-phase electrode materials. Recent studies have demonstrated that transition metal-doped ZnS systems, including Cu–ZnS, Ni–ZnS, and Co–ZnS, exhibit superior photocatalytic activity and electrochemical performance, attributed to improved light absorption, efficient charge carrier separation, and an increased density of surface-active sites.^17^ Incorporation of transition metals into ZnS effectively narrows its bandgap for enhanced visible-light absorption, while simultaneously boosting its energy storage capabilities, making these materials highly suitable for both photocatalytic degradation and supercapacitor applications.

Beyond metal hybridization, carbon-based materials such as graphitic carbon nitride (g-C_3_N_4_), graphene, reduced graphene oxide (rGO), and carbon nanotubes (CNTs) have been extensively investigated for further enhancement of ZnS-based systems.^18–20^ These carbonaceous components provide high surface area, outstanding electrical conductivity, and efficient charge transport pathways, all of which are critical to optimizing the performance of supercapacitor electrodes.^21^ The integration of carbon materials with transition metal-incorporated ZnS hybrids is anticipated to significantly improve electron transfer kinetics and ion diffusion processes, thereby enhancing charge storage and cycle stability. In photocatalytic systems, carbon-based materials can function as effective charge transport mediators, substantially suppressing recombination losses and extending the absorption range into the visible spectrum, thus elevating the overall photocatalytic efficiency of ZnS-based materials.

In light of these considerations, the present study focuses on the synthesis of g-C_3_N_4_/Cu–ZnS hybrid nanostructures as dual-functional materials for supercapacitor and photocatalytic applications. The synergistic integration of g-C_3_N_4_ with Cu-incorporated ZnS and carbon-rich conductive frameworks enhances charge storage capacity, photocatalytic efficiency, and structural integrity. The incorporation of Cu improves charge carrier mobility, while g-C_3_N_4_ prevents nanoparticle agglomeration and facilitates efficient electron transport. This study aims to develop a high-performance hybrid electrode capable of simultaneous energy storage and pollutant degradation. The core objectives include material synthesis, electrochemical evaluation, photocatalytic activity assessment, and charge transfer analysis. Overall, this work proposes a sustainable multifunctional material platform for advancing renewable energy storage and environmental remediation technologies.

## 2. Experimental procedure

### 2.1 Materials

Zinc nitrate hexahydrate (Zn(NO_3_)_2_·6H_2_O) (>99%), copper Nitrate (Cu(NO_3_)_2_·3H_2_O) (>99%), melamine (C_3_H_6_N_6_) (>95%), amoxicillin, potassium hydroxide (95%) and ethanol were purchased from Merck Chemicals and Co, Tiruchirappalli, India. All the chemicals and reagents were used as received without any further treatment. Ag/AgCl (3 M KCl) was used as reference electrode, calibrated to RHE (+0.210 V), and platinum wire served as counter electrode. Additionally, all CV and GCD data were corrected for iR-drop using the instrument's iR compensation function, ensuring accurate and reliable specific capacitance values. These details have been added to the Experimental Section of the revised manuscript.

### 2.2 Synthesis of g-C_3_N_4_ and Cu/ZnS

The g-C_3_N_4_ was prepared by the thermal polymerization method using melamine as precursor and following the procedure reported earlier.^22^ In a typical synthesis, the 5 g of melamine was ground and transferred to a ceramic crucible with a lid. The heating rate was 20 °C min^−1^ with a holding time of 2 h. After this, the solid sample was allowed to cool naturally, ground well, washed with a deionized water–ethanol mixture and dried overnight at 80 °C in an oven. The resulting g-C_3_N_4_ samples were labeled as GCN. The yield of the optimum g-C_3_N_4_ was about 45%. The hydrothermal technique was used to synthesize the Cu/ZnS hybrid. In brief, 0.1 g of zinc nitrate hexahydrate and 0.011 g of copper nitrate trihydrate were dissolved in 40 mL of deionized water, with constantly stirred. To this suspension, 0.12 g of thiourea was then added dropwise, with complete addition maintained for 30 min. The molar ratio of thiourea to metal precursors (0.12 g thiourea/1.58 mmol relative to 0.1 g Zn(NO_3_)_2_·6H_2_O/0.34 mmol). The resultant solution was transferred into a 50 mL Teflon-lined autoclave and heated to 180 °C for 12 h. After this, the product was centrifuged, thoroughly rinsed with ethanol and deionized water, and then dried overnight at 80 °C. The obtained Cu/ZnS hybrid that was produced was collected and utilized for producing the composite.

### 2.3 Synthesis of Cu/ZnS@g-C_3_N_4_

Cu/ZnS@g-C_3_N_4_ composites were prepared with varying g-C_3_N_4_ weight percentages (20%, 25%, and 30%) as follows: In this process, 0.02 g Cu/ZnS, 0.1 g g-C_3_N_4_, and 0.5 g NH_4_Cl were added into 40 mL of solvent, composed of 10 mL ethanol and 30 mL deionized water, under vigorous stirring and ultra-sonication for 1 h. The resulting mixture was then transferred into a 50 mL Teflon-lined autoclave and placed in a drying oven at 180 °C for 12 h. After the reaction, the sample was collected by centrifugation, thoroughly washed with ethanol and deionized water. The obtained product was 20 wt% g-C_3_N_4_/Cu–ZnS. Following the same procedure, Cu/ZnS@g-C_3_N_4_ composites with 25 wt% and 30 wt% of g-C_3_N_4_ were synthesized by varying the amount of g-C_3_N_4_ while keeping the other parameters constant. The obtained products of Cu/ZnS@20%, 25% and 30% of g-C_3_N_4_ were labeled as CuZnS-GCN20, CuZnS-GCN25 and CuZnS-GCN30 respectively.

### 2.4 Photocatalytic experiment

Cu/ZnS@ g-C_3_N_4_ hybrid was used as the photocatalyst in the photocatalytic degradation of the antibiotic AMX under visible light irradiation. 20 mg of photocatalyst is dispersed in 50 mL of 10 ppm of AMX in aqueous solution under sonication for 5 min. 0.1 M HCl was used to bring the pH of a 100 mL solution of amoxicillin (10 mg L^−1^) in deionized water down to the acidic level. Before exposure to UV irradiation, an adsorption–desorption equilibrium was performed by 30 min of stirring the suspension solution in the dark. The suspension was exposed to visible light irradiation using a 300 W Xenon lamp for different time durations. After light irradiation, 5 mL of the solution was taken out every 10 min, centrifuged, and its remaining concentration was determined by UV-vis absorption. The photocatalytic activities were evaluated by monitoring the changes in AMX absorption intensity at 272 nm as an indicator of AMX concentration over the duration of visible light irradiation. The degradation efficiency (%) was calculated using the equation:^23^
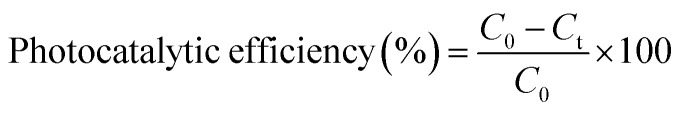
where *C*_0_ and *C*_t_ represent the initial and remaining concentrations of amoxicillin at time *t*, respectively.

### 2.5 Preparation of Cu/ZnS@g-C_3_N4 supercapacitor electrodes

The electrochemical performance of the synthesized electrodes is examined in a three-electrode electrochemical cell system, utilizing an Ag/AgCl reference electrode, a Pt counter electrode, and the synthesized electrode materials as the working electrode in a 3 M KOH solution. For the preparation of working electrodes, a homogenous slurry comprising the as-prepared Cu/ZnS@g-C_3_N_4_ composite, carbon black, and a poly-tetraflouroethylene (PTFE) binder in an 8 : 1 : 1 (w/w) ratio was mixed to make the working electrodes. The resulting slurry is applied to pre-cleaned FTO glass (1 cm × 1 cm) and subsequently dried in a hot air oven at 70 °C for 10 h. The dried electrode was subjected to additional compression at a pressure of 10 MPa to improve both electrical conductivity and mechanical adhesion. The final mass loading of the working electrode was around 1.3 mg cm^−2^. All the electrochemical characterization, such as CV, GCD, and EIS, was performed using a Biologic potentiostat (model: SP 300) workstation. The ASC device was assembled by sandwiching a Whatman 40 filter paper soaked in 3 M PVA/KOH gel electrolyte as a separator between the Cu/ZnS@g-C_3_N_4_–coated Ni foam (anode) and the activated carbon-coated Ni foam (cathode) in a Swagelok-type cell. This process is not only reproducible but also amenable to scale-up due to the absence of complex equipment, toxic solvents, or labor-intensive purification.

## 3. Results and discussion

### 3.1 Structural analysis

**Fig. 1:**
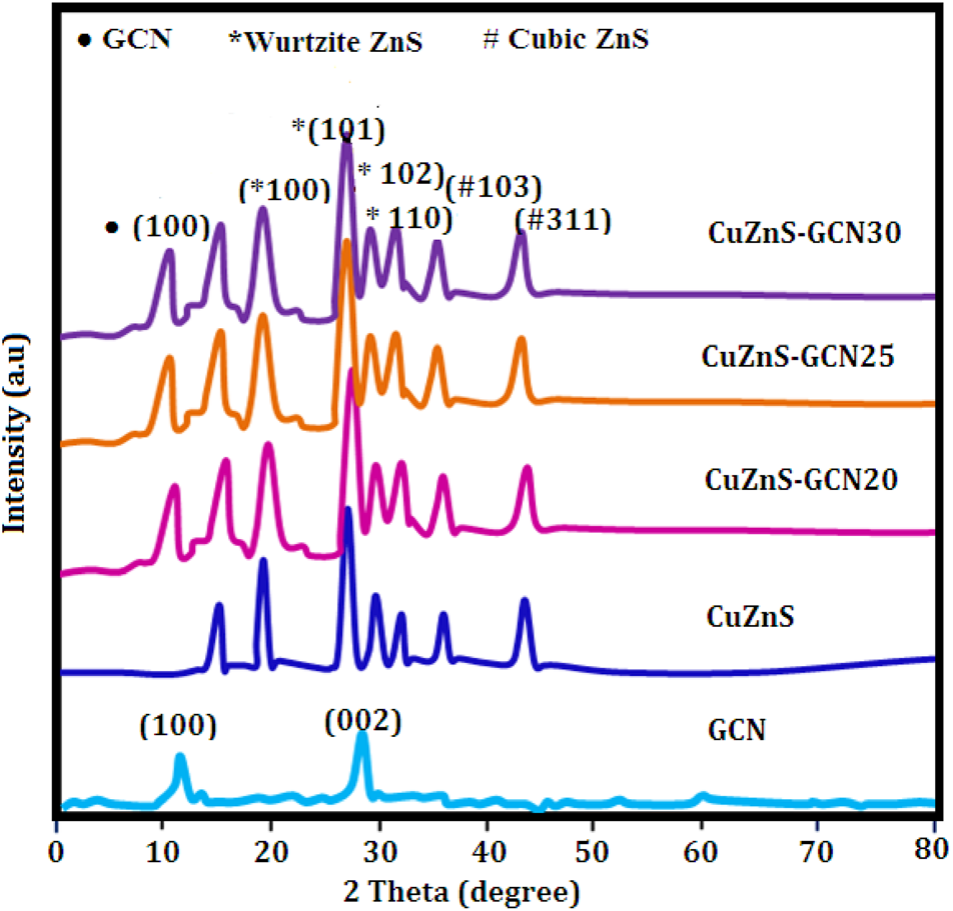
Powder XRD patterns of the prepared samples.

The X-ray diffraction (Bruker AXS D8 Advance X-ray diffractometer) was used to study the crystallinity of the samples and the obtained patterns of all the investigated samples are shown in Fig. 1. The diffraction peaks observed at 12.8° and 27.9° are indexed to (1 0 0) and (0 0 2) lattice planes of g-C_3_N_4_, respectively.^24^ These peaks represent the in-plane structural packing of the tri-*s*-triazine units and the interlayer stacking of the conjugated aromatic system, consistent with the layered graphitic structure of g-C_3_N_4_. The XRD pattern of CuZnS indicates the existence of a mixed-phase ZnS structure, incorporating both hexagonal (wurtzite) and cubic (sphalerite) phases. The diffraction peaks at 17.6°, 26.4°, 31.2°, and 33.4° correspond to the (100), (101), (102), and (110) planes of hexagonal ZnS (JCPDS No. 36-1450), whereas the peaks at 37.4° and 43.6° are associated with the (103) and (311) planes of cubic ZnS (JCPDS No. 05-0566). The simultaneous presence of both phases suggests a partial structural alteration or strain caused by the integration of Cu into the ZnS lattice. The peak at 14.2° may indicate a slight lattice distortion associated with Cu–S bonding or the emergence of Cu^+^ dopant-induced sub-structures inside the ZnS matrix.^25^ The effective hybrid formation with g-C_3_N_4_ is confirmed by the diffraction pattern of the CuZnS-GCN composites. The typical (002) peak at 27.9° is suppressed or shifted as a result of the incorporation of g-C_3_N_4_ into the CuZnS system. This phenomenon may be attributed to exfoliation effects or interfacial interactions between the CuZnS nanoparticles and the g-C_3_N_4_ nanosheets. This interaction has the potential to disrupt the stratified stacking and improve the electronic communication between components.^26^ Additionally, when the g-C_3_N_4_ concentration rises to 25 weight percent, the strength of the ZnS-related peaks, especially the (101) reflection at 26.4°, increases, indicating increased interfacial coupling and higher crystallinity. Beyond 25%, however, there is a modest decrease in peak intensity, suggesting that too much g-C_3_N_4_ may prevent crystal development or facilitate partial amorphization as a result of interfacial stress and over-saturation. The hybrid system's photocatalytic and electrochemical efficacy is enhanced by the intense interfacial interactions and observed mixed-phase structure. The g-C_3_N_4_ functions as a charge mediator and dispersing scaffold, contributing to improved charge separation and reduced recombination, while the dual-phase ZnS configuration enhances light absorption and establishes multiple charge migration pathways. In addition, Williamson–Hall (W–H) analysis was performed on the CuZnS-GCN25 sample to further investigate the crystallographic broadening effects. This was achieved by utilising multiple diffraction peaks. The observed peak broadening was attributed to both crystallite size and microstrain, as evidenced by a linear fit of β cos *θ versus* 4 sin *θ*. Upon calculation, the average crystallite size was approximately 216 nm, and the derived strain was −0.00034, which implies the presence of mild compressive lattice distortion. This strain is most likely the result of Cu loading into the ZnS lattice and the interfacial integration with g-C_3_N_4_ nanosheets. The W–H analysis confirms the presence of both nanostructuring and strain effects, which can be advantageous for improving interfacial reactivity and charge mobility in energy storage and photocatalytic applications.

### 3.2 Microstructural analysis

**Fig. 2:**
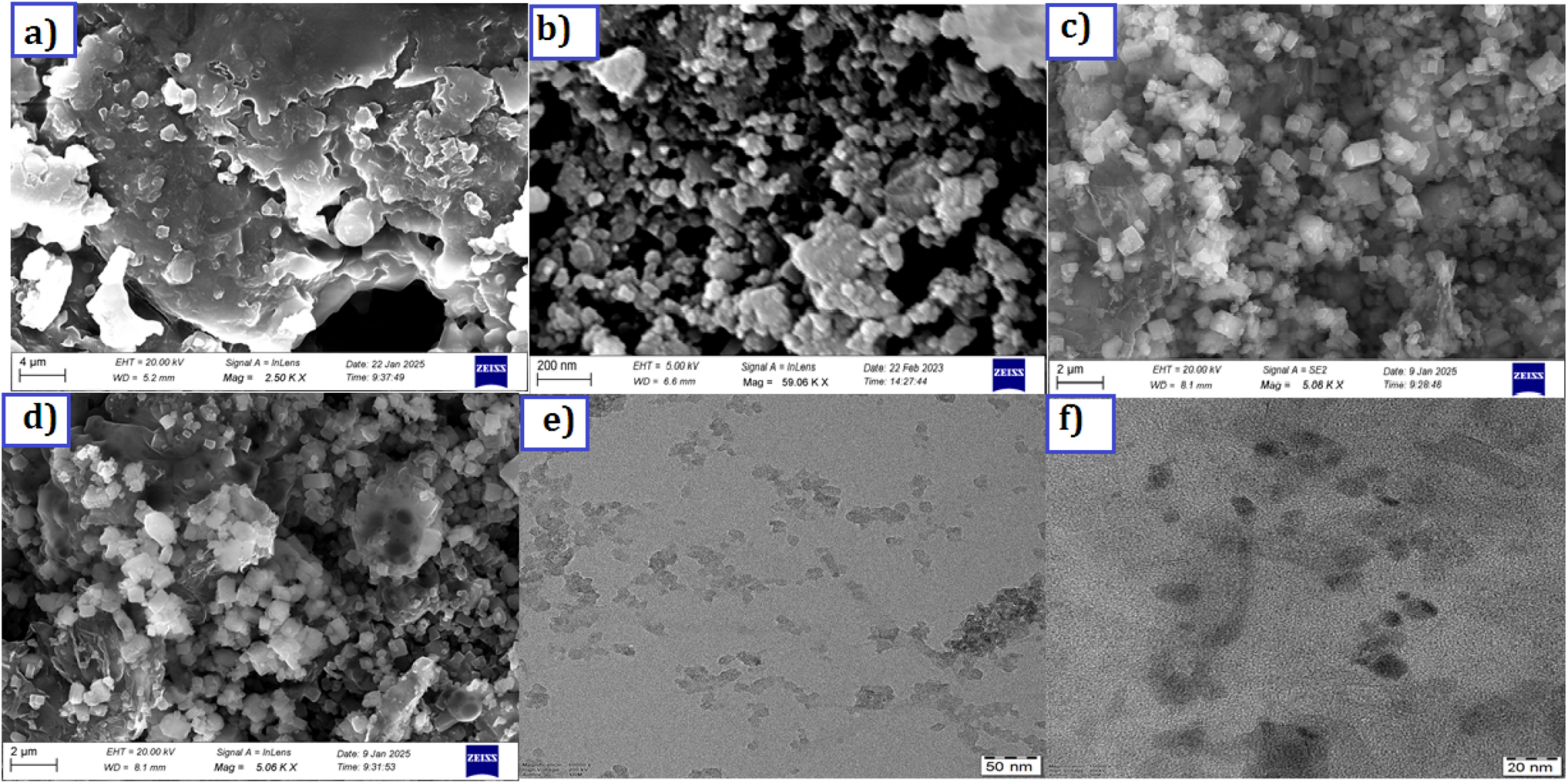
SEM micrographs of (a) GCN (b) CuZnS (c) CuZnS-GCN20 (d) CuZnS-GCN25; TEM micrographs (e) CuZnS (f) CuZnS-GCN25.

The morphology and microstructure of the Cu/ZnS@g-C_3_N_4_ hybrids were characterized by SEM and TEM techniques. Fig. 2(a–d) shows the SEM images of pure GCN, CuZnS, and CuZnS-GCN25 composite. In Fig. 2(a), the g-C_3_N_4_ exhibited a typical 2D sheet-like structure with numerous wrinkles and folds. As shown in Fig. 2(b), the Cu/ZnS nanoparticles had a nearly spherical shape with some irregularities due to structural changes caused by Cu inclusion and phase transformation. The XRD results confirmed a mix of hexagonal and cubic ZnS phases, affecting the final morphology. Fig. 2(c and d) shows that the CuZnS-GCN25 composite has a well-integrated hybrid structure with Cu/ZnS nanoparticles uniformly attached onto the g-C_3_N_4_ sheets. Adding Cu/ZnS to the g-C_3_N_4_ surface reduced wrinkles and folds due to their interaction. Fig. 2(e) shows a TEM image of CuZnS, which has a roughly spherical shape with slight imperfections, validating the SEM results. The well-defined lattice fringes in the high-resolution TEM (HRTEM) image indicate the crystalline character of the CuZnS nanoparticles, indicating the existence of mixed hexagonal and cubic phases as detected by the XRD study. Moreover, in Fig. 2(f), the TEM image of the CuZnS-GCN25 composite shows a homogeneous dispersion of Cu/ZnS nanoparticles over the GCN sheets. CuZnS and GCN exhibit high interfacial interaction, which allows for efficient charge transfer and separation.

### 3.3 FTIR, UV analysis

**Fig. 3:**
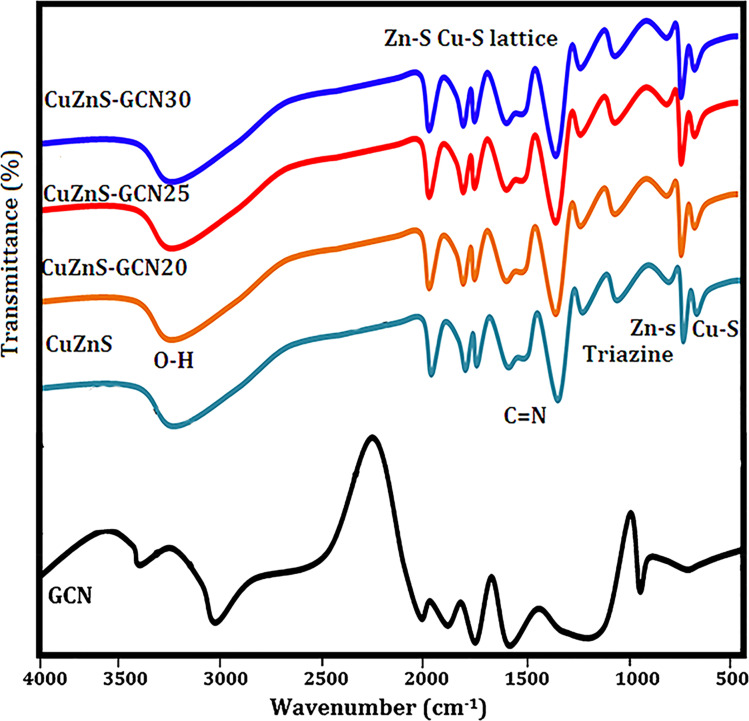
FTIR spectra of the prepared samples.

**Fig. 4:**
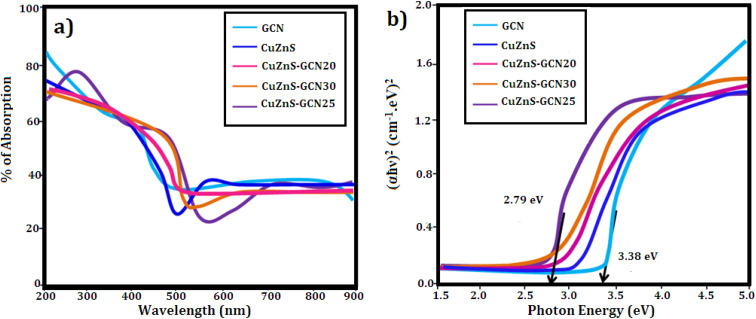
(a) UV-DRS spectra of g-C_3_N_4_, CuZnS, CuZnS-GCN20, CuZnS-GCN25 and CuZnS-GCN30 samples. (b) Tauc plot of band gap calculation.

Fig. 3 shows the FTIR spectrum of GCN, CuZnS, and CuZnS-GCN hybrid with varying amounts of GCN. Pristine GCN has IR peaks that are similar to those seen in earlier findings. Fig. 3 illustrates that the distinctive peaks at 3000–3500 cm^−1^ and 810 cm^−1^ may be attributed to the vibrations of N–H bonds, and s-triazine subunits.^27^ Moreover, the bands in the 1200–1650 cm^−1^ regions correspond to the typical stretching modes of C–N heterocycles. The FTIR spectrum of CuZnS validates its structural composition *via* distinctive vibrational modes. The extensive absorption band between 3400 and 3500 cm^−1^ is attributed to O–H stretching vibrations, signifying the presence of surface–OH groups.^28^ The peak at around 1630 cm^−1^ is ascribed to the bending vibrations of H–O–H from adsorbed water. The prominent peaks detected between 500–700 cm^−1^ are attributed to the Zn–S and Cu–S stretching vibrations.^29^ The FT-IR spectra of CuZnS-GCN composites exhibited distinctive peaks of GCN, with peak intensity increasing as the GCN concentration reached an optimal 25%, signifying greater interaction among the components. The unique peaks related to both Cu/ZnS and GCN proved the formation of well-integrated hybrids between them. The optical properties of GCN, CuZnS, CuZnS-GCN 20, CuZnS-GCN 25, and CuZnS-GCN 30 were analyzed using UV-vis diffuse reflectance spectra (Fig. 4(a)). The figure illustrates that GCN exhibited a distinct absorption band between 300 and 500 nm, with an absorption edge approximately at 465 nm, indicating that the sample had a narrow band gap. This absorption spectrum is comparable to the reported spectra for g-C_3_N_4_.^30^ Meanwhile, CuZnS retains its UV-induced response because of the wide band gap with an absorption edge below 500 nm. The CuZnS-GCN composite exhibits an extension of the absorption edge into the range of visible light. The absorption intensity in the visible light spectrum increased with the rising GCN content in the CuZnS-GCN composites, reaching a maximum at 25% GCN. This enhancement may be attributed to the robust interfacial interaction between Cu/ZnS and g-C_3_N_4,_ resulting in improved light-harvesting capacity and efficient charge transfer. For calculating the optical bandgap energy for the samples Tauc equation was used.^31^ From Fig. 4(b), it could be seen that the band gaps of GCN, CuZnS, CuZnS-GCN20, and CuZnS-GCN25 were calculated to be 3.38 eV, 3.15 eV, 2.96 eV, 2.79 eV, and 2.85 eV, respectively. Compared to all other samples, the CuZnS-GCN25 composite (at 25% GCN content) showed a narrower band gap, which indicates that the existence of strong interfacial interactions between CuZnS and g-C_3_N_4_ facilitated effective charge transfer and enhanced visible-light absorption.

### 3.4 BET and XPS analysis

**Fig. 5:**
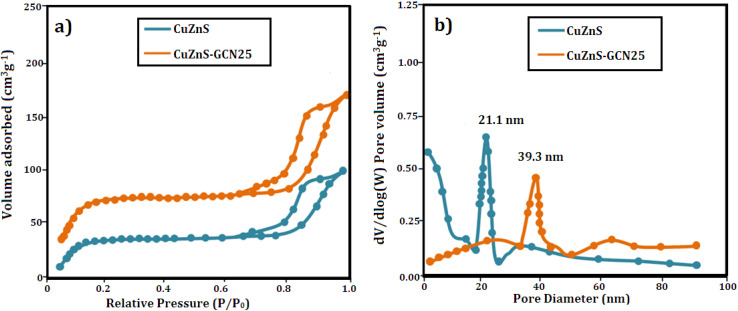
(a) N_2_ adsorption–desorption isotherm of CuZnS and CuZnS-GCN25 (b) BJH plot for pore size calculation.

**Fig. 6:**
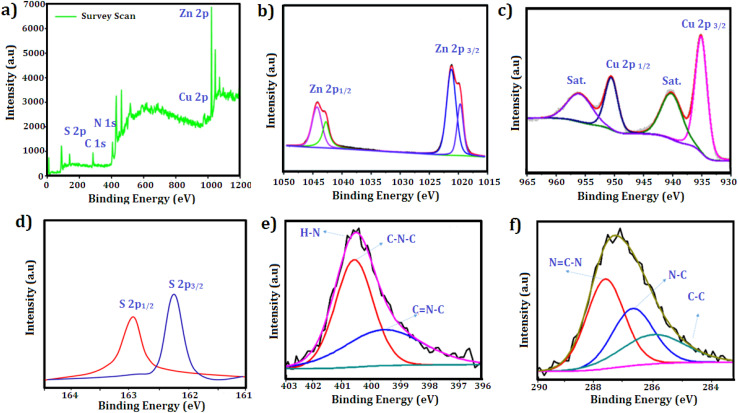
XPS spectra results of CuZnS-GCN25 (a) survey scan spectra; core level scan (b) Zn 2p (c) Cu 2p (d) S 2p (e) N 1s (f) C 1s.

The N_2_ adsorption–desorption isotherms depicted in Fig. 5(a) were utilized to analyze the BET specific surface areas of CuZnS and CuZnS-GCN25 composites. The results indicated that both materials exhibited type IV isotherms *via* the hysteresis loop, suggesting mesoporous structures.^32^ It was also observed that the inflection point shifted to lower relative pressure in the order of CuZnS > CuZnS-GCN25, demonstrating the contraction of the pore size distribution. The pore size distribution was determined by using the BJH method and shown in Fig. 5(b).^33^ The specific surface areas for CuZnS and CuZnS-GCN25 hybrids are 84.22 m^2^ g^−1^ and 148.16 m^2^ g^−1^, respectively, whereas the average pore diameters for CuZnS and CuZnS-GCN25 are 21.1 nm and 39.30 nm. The surface area of CuZnS-GCN25 is almost 1.6 times more prominent than that of CuZnS sample. This result indicates that CuZnS-GCN25 possesses a greater number of active sites and enhanced absorption and degradation capabilities compared to bare CuZnS. The surface chemical compositions of the optimized g-C_3_N_4_ integrated CuZnS hybrid were analyzed using XPS analysis and the results are depicted in Fig. 6(a–f). The survey spectrum of the Cu, Zn, S, C and N elements is displayed in Fig. 6(a), where the four bands at 932 eV, 1021 eV, 162 eV, 284 eV, and 398 eV correspond to Cu 2p, Zn 2p, S 2p, C 1s, and N 1s, respectively. Fig. 6(b) illustrates that the Zn 2p core level spectra for the CuZnS-GCN25 composite have two peaks at 1021.8 eV and 1044.6 eV, corresponding to Zn 2p_3/2_ and Zn 2p_1/2_, respectively.^34^ The spin–orbit splitting of Zn 2p_1/2_ and Zn 2p_3/2_ is 22.8 eV, a typical feature of ZnS. The Cu 2p high-resolution spectrum displays two separate peaks at 932.4 eV and 952.3 eV, which correspond to Cu 2p_3/2_ and Cu 2p_1/2_, respectively (Fig. 6(c)). Furthermore, the absence of pronounced satellite peaks signifies that Cu mainly exists in the Cu^+^ state, implying efficient interaction with the ZnS lattice.^35^ The high-resolution S 2p spectra depicted in Fig. 6(d) indicate binding energies at 162.3 eV and 163.5 eV, attributed to S 2p_3/2_ and S 2p_1/2_, respectively, indicating the existence of sulphide species in the CuZnS@g-C_3_N_4_ composite. As shown in Fig. 6(e), the N 1s core level spectrum was deconvoluted into three peaks centered at 398.8 eV, 399.4 eV, and 400.9 eV, corresponding to sp^2^ hybridized C=N–C bonds in the graphitic g-C_3_N_4_ framework, bridging N (C–N–C) atoms, and terminal N–H_*x*_ groups, respectively.^36^ Moreover, three distinct peaks, 284.6 eV (C–C), 286.3 eV (N–C–N), and 288.3 eV (N=C–N), can be distinguished in the case of C 1s (Fig. 6(f)), respectively.^37^ These findings lead us to the conclusion that in the CuZnS-GCN25 sample, g-C_3_N_4_ and CuZnS coexist. Therefore, the XPS results once more confirm the formation of the CuZnS/g-C_3_N_4_ composite.

### 3.5 Electrochemical performance analysis in three electrode system

#### 3.5.1 Cyclic voltammetry

**Fig. 7:**
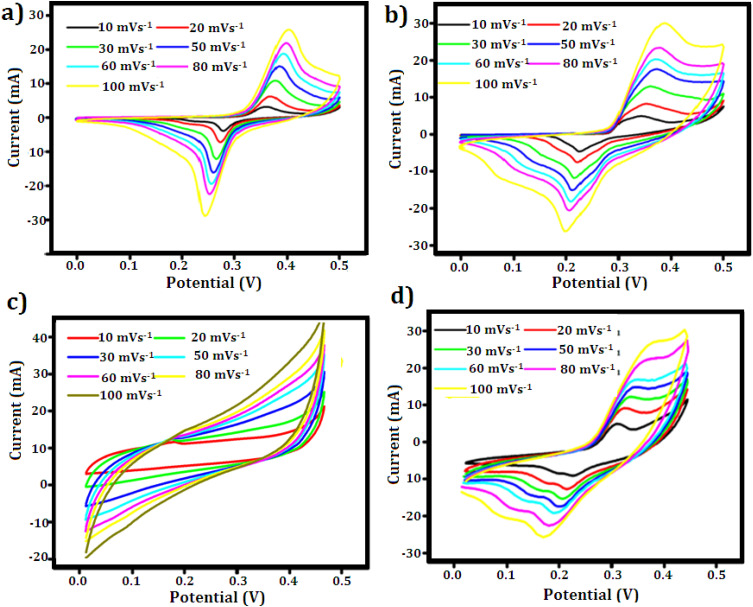
CV curves of (a) CuZnS (b) CuZnS-GCN20 (c) CuZnS-GCN25 (d) CuZnS-GCN30 electrodes recorded at different scan rates in 3 M KOH electrolyte.

**Fig. 8:**
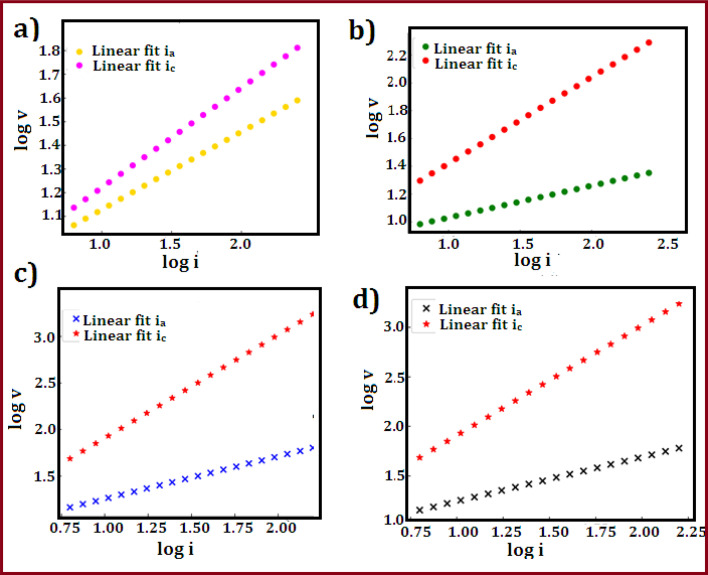
Logarithmic plot of peak current (*I*p) *vs.* scan rate (*v*) for (a) CuZnS, (b) CuZnS-GCN20, (c) CuZnS-GCN25 and (d) CuZnS-GCN30 electrodes.

Cyclic voltammetry (CV) and galvanostatic charge–discharge (GCD) investigations in a three-electrode system with 3 M KOH as the electrolyte were used to assess the electrochemical performance of the fabricated electrodes. At first, the charge storage mechanism and redox behavior of the CuZnS, CuZnS-GCN20, CuZnS-GCN25 and CuZnS-GCN30 electrodes were examined using CV analysis at various scan rates. As shown in Fig. 7(a–d), the CV profiles exhibit distinct redox peaks, with a progressive increase in current response upon the incorporation of GCN, confirming an enhancement in the electrochemical activity. Among all the composites that were examined, CuZnS-GCN25 notably exhibits the greatest current response, indicating its better electrochemical behaviour. CuZnS-GCN25 and CuZnS-GCN30 show a downward trend, whereas CuZnS and CuZnS-GCN20 show an upward trend. This shift in peak orientation suggests that the process is shifting from mostly capacitive to faradaic-controlled charge storage. The main causes of this change are the more electroactive sites and improved charge transport channels made possible by the addition of GCN.^38^ To further quantify the charge storage properties, the specific capacitance (C, in F g^−1^) was calculated from the CV curves using the following equation:^39^
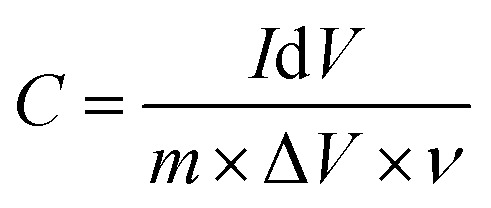
where *m* is the mass of the active material (g), Δ*V* is the potential range, *v* is the scan rate (V s^−1^), d*V* is the potential window (V), and *I* stand for the current (A). Table 1 summarizes the calculated capacitance values for each of the four samples at various scan rates. The capacitance values show that CuZnS-GCN25 has the highest charge storage ability, followed by CuZnS-GCN30, CuZnS-GCN20 and then CuZnS. This clearly shows that CuZnS-GCN25 performs very well. Specifically, at the scan rate of 10 mV s^−1^, CuZnS-GCN25 has a capacitance of 786 F g^−1^, which is much higher than CuZnS (455 F g^−1^). This improvement is due to the combined effect of CuZnS and GCN working together. Although CuZnS-GCN30 also has high capacitance, it is slightly lower than CuZnS-GCN25 (715 F g^−1^). This suggests that adding too much GCN might increase resistance or slow down ion movement, reducing performance.^40^ Moreover, the CV curves demonstrate a notable rise in current response as scan rates increase, indicating effective charge storage and transfer across all samples. Distinct variations in peak shifts and current response trends highlight the differences in charge storage mechanisms across the electrodes. As shown in Fig. 7 (a–d), the CV profiles for CuZnS and CuZnS-GCN20 exhibit a relatively symmetric nature, accompanied by a slight increase in current at elevated scan rates, indicating a primarily capacitive charge storage mechanism. The almost linear rise in current with scan rate and the slight peak separation suggest that charge storage is mainly controlled by electrical double-layer capacitance (EDLC), where charge is established at the electrode–electrolyte interface with minimal faradaic effects.^41^ In comparison, CuZnS-GCN25 and CuZnS-GCN30 display distinct redox peaks, with a significant shift in peak positions towards higher potentials as the scan rate is elevated. This behavior indicates a shift towards a more faradaic charge storage mechanism, wherein redox reactions play a crucial role in enhancing the overall charge storage capacity. The clearly defined redox peaks in CuZnS-GCN25, combined with its peak current response across all scan rates, validate its exceptional electrochemical kinetics and effective charge transfer pathways. Additionally, the examination of peak current (*I*p) against the scan rate (*v*) through the power law equation = *I*p = *av*^*b*^ reveals more information about the charge storage mechanism (Fig. 8(a–d)). The extracted *b*-value of 0.80 for CuZnS-GCN25 suggests that both capacitance and diffusion-controlled redox reactions contribute to the charge storage. This coexistence enables high power density and energy storage effectiveness. In comparison, CuZnS and CuZnS-GCN20 have lower *b* values, in the region of 0.6, indicating their stronger dependence on diffusion-limited charge storage, which is typical of materials with few active electrochemical sites. CuZnS-GCN30 has a *b*-value slightly lower than CuZnS-GCN25, indicating that although this material exhibits strong redox activity, excessive GCN content increases resistance to ion diffusion. Moreover, the peak-to-peak separation increases at higher scan rates, especially with CuZnS-GCN25 and CuZnS-GCN30, which indicates increased charge, transfer resistance at higher scan rates. This is further supportive that CuZnS-GCN25 has the most efficient charge transfer kinetics and so allows maximum charge storage and release with minimum energy loss. The exceptional electrochemical performance of CuZnS-GCN25 is due to the optimum composition of CuZnS and GCN, ensuring improved conductivity, a high density of electroactive sites, and efficient charge transport pathways.

**Table 1:** Calculated specific capacitance of fabricated electrodes from CV data

Scan rate (m Vs^−1^)	Spefic capacitance from CV			
	CuZnS	CuZnS-GCN 20	CuZnS-GCN 25	CuZnS-GCN 30
10	455 F g^−1^	624 F g^−1^	786 F g^−1^	715 F g^−1^
20	441 F g^−1^	610 F g^−1^	771 F g^−1^	709 F g^−1^
30	430 F g^−1^	601 F g^−1^	760 F g^−1^	698 F g^−1^
50	424 F g^−1^	594 F g^−1^	748 F g^−1^	681 F g^−1^
60	411 F g^−1^	579 F g^−1^	735 F g^−1^	665 F g^−1^
80	403 F g^−1^	561 F g^−1^	721 F g^−1^	651 F g^−1^
100	389 F g^−1^	552 F g^−1^	705 F g^−1^	643 F g^−1^

#### 3.5.2 GCD analysis

**Fig. 9:**
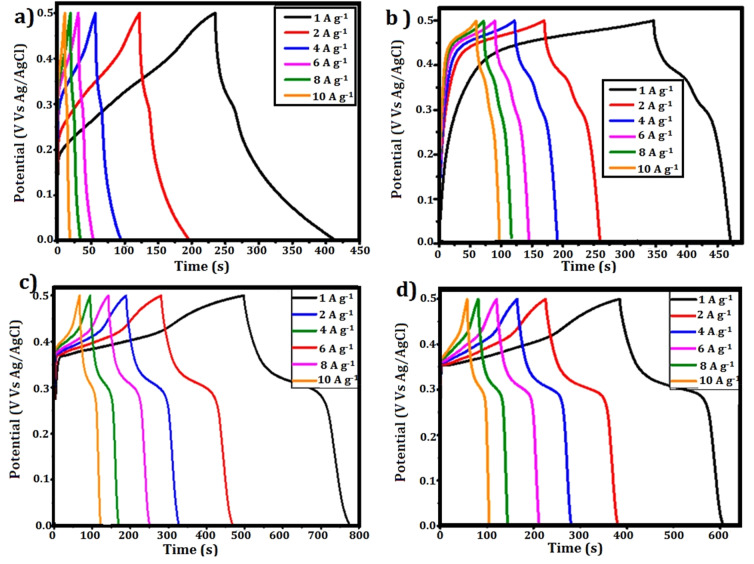
GCD curves of (a) CuZnS (b) CuZnS-GCN20 (c) CuZnS-GCN25 and (d) CuZnS-GCN30 electrodes at various current densities.

**Fig. 10:**
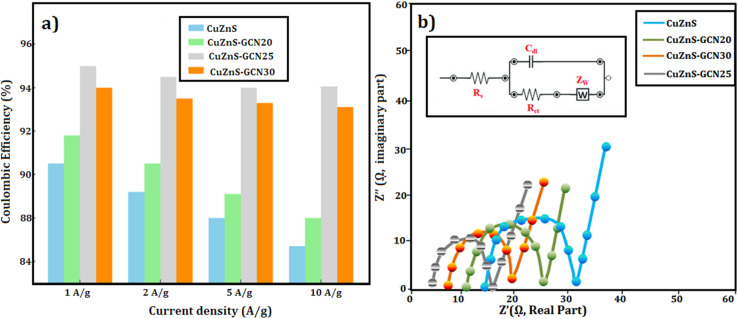
(a) Coulombic efficiency (%) *vs.* current density (b) Nyquist plot.

To assess the charge storage capabilities and rate performance of the synthesised electrodes, GCD experiments were carried out in a three-electrode system with 3 M KOH as the electrolyte. Fig. 9(a–d) shows the GCD profiles for CuZnS, CuZnS-GCN20, CuZnS-GCN25, and CuZnS-GCN30 at current densities of 1 A g^−1^ to 20 A g^−1^. The measured charge–discharge curves have nonlinear properties, demonstrating the presence of both capacitive and faradaic charge storage processes. Notably, the CuZnS-GCN25 electrode has the longest charge–discharge duration, indicating greater charge storage capacity, which is consistent with the CV analysis. The specific capacitance values were determined using the GCD curves according to the following equation:^42^
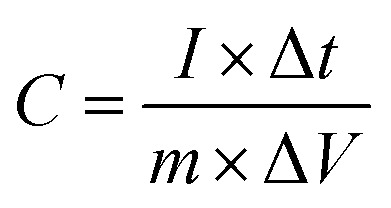
where *I* is the discharge current (A), Δ*t* is the discharge time (s), *m* is the electrode's active mass (g), and Δ*V* is the potential window. Table 2 summarizes the determined specific capacitance values for different current densities. As listed in Table 2, at 1 A g^−1^, CuZnS-GCN25 has a capacitance of 603 F g^−1^, which is significantly higher than CuZnS (312 F g^−1^), CuZnS-GCN20 (448 F g^−1^), and CuZnS-GCN30 (491 F g^−1^). CuZnS-GCN25's increased capacitance is due to its optimized composition, which allows for faster charge transfer and increases the number of redox-active sites. As the current density increases from 1 to 10 A g^−1^, all electrodes experience a smaller drop in specific capacitance due to ion diffusion restrictions at higher current densities. However, at 10 A g^−1^, CuZnS-GCN25 retains 83% of its original capacitance, indicating outstanding rate capability. This enhanced electrochemical behaviour verifies the effective electron transport routes and synergistic interaction of CuZnS and GCN, which results in increased charge storage performance. The efficacy of charge storage and retrieval in supercapacitors is critically determined by the coulombic efficiency (*η*) parameter.^43^ It is determined by the ratio of discharge time to charge time at a constant current density, which indicates that the materials are highly stable and reversible. CuZnS-GCN25 has the highest coulombic efficiency of 94.2% among all electrodes, suggesting that there is minimal energy loss during charge–discharge cycling (Fig. 10(a)). This implies that the redox reactions are extremely reversible and that the electrochemical stability is exceptional. An increase in charge transfer resistance caused by excess GCN content may have contributed to the marginally lower efficacy of CuZnS-GCN30 (93.1%), which may prevent ion diffusion. The coulombic efficacy of all electrodes decreases gradually as the current density increases, as a result of limited ion diffusion and an increased polarization effect at higher charge–discharge rates. Nevertheless, CuZnS-GCN25 exhibits an exceptional charge retention capability and reversibility, as evidenced by its high coulombic efficiency of 94.2% at 10 A g^−1^. The high coulombic efficiency values (>90%) across all electrodes demonstrate the produced materials' remarkable electrochemical stability and low internal resistance, making them ideal candidates for real-world supercapacitor applications.

**Table 2:** Calculated specific capacitance of fabricated electrodes from GCD data

Current density (A g^−1^)	Spefic capacitance from GCD			
	CuZnS	CuZnS-GCN 20	CuZnS-GCN 25	CuZnS-GCN 30
1	312 F g^−1^	448 F g^−1^	603 F g^−1^	491 F g^−1^
2	297 F g^−1^	425 F g^−1^	584 F g^−1^	475 F g^−1^
4	272 F g^−1^	403 F g^−1^	570 F g^−1^	454 F g^−1^
6	265 F g^−1^	389 F g^−1^	541 F g^−1^	431 F g^−1^
8	254 F g^−1^	370 F g^−1^	523 F g^−1^	413 F g^−1^
10	235 F g^−1^	355 F g^−1^	505 F g^−1^	399 F g^−1^

#### 3.5.3 EIS analysis

EIS measurements were conducted within a frequency range of 100 kHz to 0.01 Hz, utilizing an applied AC perturbation of 5 mV, to provide a comprehensive understanding of the charge transfer and internal resistance of the manufactured electrodes. Fig. 10(b) illustrates the Nyquist plots for CuZnS, CuZnS-GCN20, CuZnS-GCN25, and CuZnS-GCN30, exhibiting a characteristic semicircle in the high-frequency domain and a linear extension in the low-frequency domain. The intercept on the real axis (*Z*′) at elevated frequencies signifies the analogous series resistance (*R*_s_), linked to the internal resistance of the electrode material and electrolyte.^44,45^ The equivalent fitted circuit is also elaborated in the inset. The semicircular diameter represents the charge transfer resistance (*R*_ct_) at the electrode–electrolyte interface, whereas the Warburg impedance (*Z*_w_), depicted by the inclined line in the low-frequency zone, signifies ion diffusion resistance. The calculated values indicate that CuZnS-GCN25 has the lowest Rct value of 4.2 Ω cm^2^, which is much lower than that of CuZnS (15.8 Ω cm^2^), CuZnS-GCN20 (9.5 Ω cm^2^), and CuZnS-GCN30 (6.1 Ω cm^2^). The *R*_s_ was found to obey a similar trend, with CuZnS-GCN25 having the lowest 1.2 Ω cm^2^ smaller as compared to CuZnS (5.8 Ω cm^2^), CuZnS-GCN20 (2.5 Ω cm^2^), and CuZnS-GCN30 (2.1 Ω cm^2^). The reduced charge transfer resistance in CuZnS-GCN25 validates its exceptional electrical conductivity and effective charge transport routes, hence improving electrochemical performance. The sharper slope in the low-frequency zone for CuZnS-GCN25, relative to other electrodes, indicates accelerated ion diffusion kinetics, essential for sustaining high capacitance at elevated scan rates.

### 3.6 Electrochemical performance analysis in the two-electrode system

**Fig. 11:**
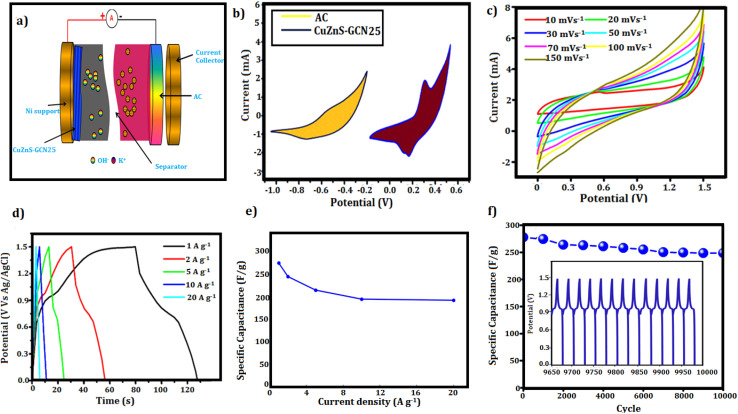
(a) Schematic representation of the asymmetric supercapacitor device assembled using CuZnS-GCN25 as the electrode material (b) CV curves of CuZnS-GCN25 and activated carbon at a fixed scan rate of 10 mVs^−1^ (c) CV curve of fabricated ASC with different scan rates (10–100 m Vs^−1^) (d) GCD curve of fabricated ASC with different current densities (1–10 Ag^−1^) (e) specific capacitance *vs.* current density plot for CuZnS-GCN25 (f) cycling stability test of CuZnS-GCN25 over 10 000 charge–discharge cycles at 10 A g^−1^.

To determine the practical accessibility of the optimized CuZnS-GCN25 electrode in a super capacitor device (Fig. 11(a)), its electrochemical performance was evaluated in a two-electrode system with 3 M KOH as the electrolyte. CV measurements of the g-C_3_N_4_/Cu–ZnS hybrid electrode were carried out at multiple potential windows and scan rates. Based on these experiments, the potential window of 0 to 0.5 V was optimized and used for all CV results of the individual electrode. Similarly, activated carbon is generally tested in a potential window of 0 to −1 V, which was followed in our tests. Fig. 11(b) represents the CV profiles of both electrodes in a three-electrode configuration. These measurements helped determine that the most suitable operating range for the assembled device is 0 to 1.5 V. The Fig. 11(c) displays the CV curves of the CuZnS-GCN25-based symmetric device obtained at different scan speeds. The form of the CV curves is well-retained with increased scan rates, showing strong rate capability and the existence of both electric double-layer capacitance and faradaic contributions. GCD study was subsequently carried out at current densities ranging from 1 A g^−1^ to 20 A g^−1^ to further evaluate the charge storage ability of CuZnS-GCN25 in the two-electrode system, as shown in Fig. 11(c). As demonstrated in Fig. 11(d), the charge–discharge curves reveal a characteristic non-linear nature, demonstrating the synergistic coupling of capacitive and faradaic processes. CuZnS-GCN25 displayed the longest charge–discharge duration, corresponding with the CV data, hence proving its good electrochemical performance. The specific capacitance values determined from the GCD curves are displayed in Fig. 10(e), with CuZnS-GCN25 having a high capacitance of 275 F g^−1^ at 1 A g^−1^. Although capacitance declines significantly at increasing current densities due to diffusion restrictions, it maintains around 70% of its original capacitance at 20 A g^−1^, demonstrating remarkable rate capability. The synergistic interaction between CuZnS and GCN promotes quick charge transfer and increases the availability of redox-active sites, resulting in improved electrochemical performance. CuZnS-GCN25's cycling stability was further tested using continuous charge–discharge cycling at a current density of 10 A g^−1^ for 10 000 cycles, as shown in Fig. 11(f). Interestingly, the electrode retains 92.5% of its original capacitance after repeated cycling, demonstrating its exceptional endurance. The high coulombic efficiency of roughly 98% during the cycle test demonstrates that there is little energy loss during charge–discharge operations. The extraordinary stability is due to the strong interfacial connection between CuZnS and GCN, which inhibits structural deterioration and guarantees long-term performance dependability. Overall, the electrochemical testing of CuZnS-GCN25 in a two-electrode system reveals its exceptional charge storage properties, high-rate capability, great cycle stability, and low internal resistance. The optimized potential window of 1.6 V enables more energy storage, while the combination of capacitive and faradaic charge storage techniques improves total charge storage efficiency. CuZnS-GCN25 has great specific capacitance retention at high current densities and outstanding long-term stability, making it a potential electrode material for practical super capacitor applications.

**Fig. 12:**
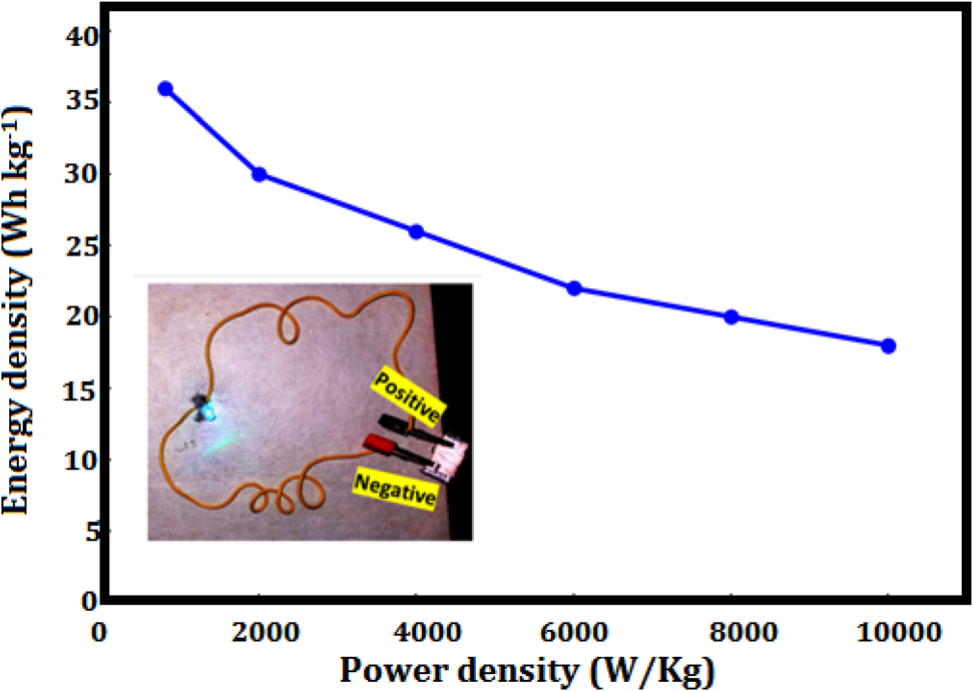
Ragone plot depicting the energy density *vs.* power density relationship for CuZnS-GCN25; figure (inset) practical demonstration of the CuZnS-GCN25-based supercapacitor device powering a green LED.

The electrochemical performance of CuZnS-GCN25 was compared with previously published Cu-based and Zn-based sulfide supercapacitor electrodes, as detailed in Table 3.^46–54^ The specific capacitance of CuZnS-GCN25 (603 F g^−1^ at 1 A g^−1^) was much greater than that of Cu-based CuI/g-C_3_N_4_/Ni foam (318 F g^−1^) and Zn-based ZnO/ZnS/g-C_3_N_4_ (152–596 F g^−1^) sulfides, revealing its better charge storage capabilities. Additionally, the long-term cycling stability of CuZnS-GCN25 was confirmed by its good capacitance retention (92.5% after 10 000 cycles), which exceeded the reported 82% (Cu-based) and 85–90% (Zn-based) after fewer cycles. The low charge transfer resistance (3.5 Ω cm^2^) and excellent coulombic efficiency (∼98%) further establish CuZnS-GCN25 as an efficient and stable electrode material for next-generation supercapacitor applications. The extensive potential window (1.6 V) facilitates increased energy density, rendering it a compelling option for hybrid supercapacitors and portable electronic devices. Furthermore, the exceptional rate capability and prolonged durability render it appropriate for grid storage applications, regenerative braking systems, and wearable electronics, where rapid charge and discharge characteristics are essential. The Ragone plot (Fig. 12) depicts the energy density *vs.* power density relationship, which is an important parameter for determining the suitability of supercapacitors for real-world applications. CuZnS-GCN25 has an outstanding energy density of 36 W h kg^−1^ and a power density of 800 W kg^−1^, far exceeding traditional carbon-based supercapacitors. At high power densities (∼10 kW kg^−1^), CuZnS-GCN25 maintains an energy density of ∼18 W h kg^−1^, indicating good power-energy balance. These results are comparable to commercial supercapacitors and hybrid capacitive devices, demonstrating CuZnS-GCN25's promise as an efficient energy storage material. The inset photograph (Fig. 12, inset) shows how the CuZnS-GCN25-based supercapacitor device can be used in real life. Furthermore, it shows that the device can power a green LED, which is direct proof of its usefulness in the real world. The bright LED light means that the device can provide enough power, even after a short charging session. This real-life example not only supports the electrochemical data from CV and GCD measurements, but it also shows how the material could be used in compact and adaptable energy storage systems. Such functionality aligns with commercial expectations for compact power sources, confirming CuZnS-GCN25's viability for next-generation supercapacitor applications. Overall, CuZnS-GCN25's outstanding electrochemical characteristics, large energy storage capacity, and consistent cycling performance position it as a potential material for next-generation energy storage devices that bridge the gap between existing supercapacitors and batteries.

**Table 3:** Comparison of performance of fabricated CuZnS-GCN25 with previous reports

Electrode	Electrolyte	Specific capacitance	Cyclic stability (% of retention)	Specific energy	References
ZnS/ZnO/g-C_3_N_4_	3 M Na_2_SO_4_	152 F g^−1^	5000 cycle (90%)	11.8 W h kg^−1^	46
ZnS/ZnO/g-C_3_N_4_	1M NaClO_4_	596 F g^−1^	1150 cycles (85%)	—	47
CuI/g-C_3_N_4_/Ni foam	1 M Na_2_SO_4_	318 F g^−1^	3000 cycles (82%)	34.2 W h kg^−1^	48
ZnO/g-C_3_N_4_	1 M Na_2_SO_4_	146 F g^−1^	5000 cycles (85%)	38.8 W h kg^−1^	49
CuS/rGO	6 M KOH	587 F g^−1^	2000 cycles (95.0%)	43 W h kg-^1^	50
Cu doped ZnS	3 M KOH	468 F g^−1^	5000 cycles (89%)	—	51
ZnS/CuSe_2_	3 M KOH	95 F g^−1^	8000 cycles (81.0%)	38 W h kg^−1^	52
CuS/ZnS/sodium alginate/rGO	0.5 M K_2_SO_4_	992 F g^−1^	1000 cycles (90.0%)	2.05 W h Kg^−1^	53
Zn_0.25_Cu_0.75_S	1 M KOH	2935 F g^−1^	10000 cycles (91%)	38.6 W h kg^−1^	54
CuZnS/g-C_3_N_4_	3 M KOH	786 F g^−1^	1000 cycles (92.5%)	36 W h kg^−1^	This work

### 3.7 Photocatalytic performance analysis

**Fig. 13:**
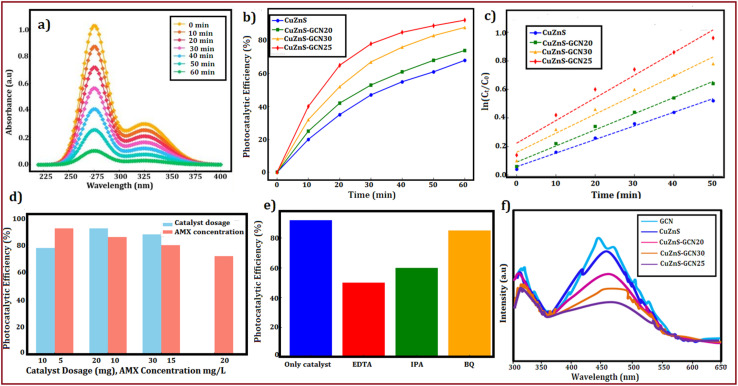
(a) UV-visible absorption spectra of AMX degradation under visible light irradiation using CuZnS-GCN25 (b) comparison of AMX degradation efficiency of prepared samples (c) pseudo-first-order kinetic plots for AMX degradation using g-C_3_N_4_, CuZnS, CuZnS-GCN20, CuZnS-GCN25 and CuZnS-GCN30 samples (d) effect of catalyst dosage (10 mg–30 mg) and initial AMX concentration (5–20 mg L^−1^) on degradation efficiency (e) radical trapping experiments (f) PL spectra of g-C_3_N_4_, CuZnS, CuZnS-GCN20, CuZnS-GCN25 and CuZnS-GCN30 samples.

**Fig. 14:**
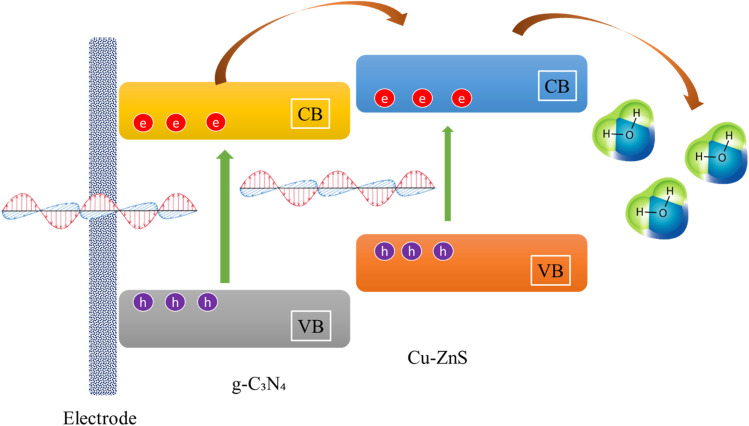
A schematic energy band diagram showing the charge transfer between Cu–ZnS and g-C_3_N_4_ and photocatalytic mechanisms.

The photocatalytic activity of the g-C_3_N_4_/Cu–ZnS hybrid nanostructure was investigated by measuring amoxicillin (AMX) degradation under visible light irradiation. AMX, a commonly used β-lactam antibiotic, frequently occurs in wastewater due to its widespread usage in human and veterinary medicine. The persistence of AMX in water sources constitutes a serious environmental danger, resulting in the growth of antibiotic-resistant bacteria, disturbance of aquatic ecosystems, and possible harm to human health. The degradation efficiency of AMX was determined by measuring its distinctive absorption peak at 272 nm during various irradiation periods. The UV-visible absorption spectra (Fig. 13(a)) show a gradual decline in the AMX peak strength with increasing irradiation period, demonstrating the antibiotic's continuing breakdown. Cu/ZnS@g-C_3_N_4_ demonstrated much better photocatalytic effectiveness than pure ZnS and g-C_3_N_4_, with a maximum degradation efficiency of 92.4% after 60 min of visible light irradiation (Fig. 13(b)). Cu doping and g-C_3_N_4_ inclusion work together to improve charge separation and limit recombination of photogenerated electron–hole pairs, resulting in enhanced photocatalytic activity.^55^ To investigate photocatalytic kinetics, the degradation rate was analysed using the pseudo-first-order kinetic model, represented by the equation:^56^
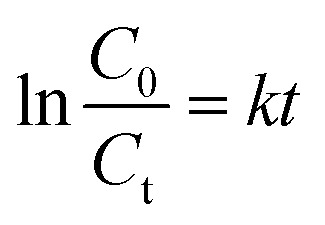
where *k* is the rate constant, and *C*_0_ and *C*_t_ are the initial and final AMX concentrations at time *t*, respectively. The kinetic curve (Fig. 13(c)) shows a linear relationship, showing that the degradation occurs using pseudo-first-order kinetics. Cu/ZnS@ g-C_3_N_4_ has a much higher rate constant (0.029 min^−1^) than pure ZnS (0.012 min^−1^) and g-C_3_N_4_ (0.018 min^−1^), indicating the hybrid material's improved photocatalytic performance. Additionally investigated were the effects of catalyst dose and starting AMX concentration. A larger degradation rate resulted from increasing the catalyst loading from 10 mg to 30 mg, as seen in Fig. 13(d). This suggests that there were more active sites accessible for photocatalysis. However, because of increased light scattering and decreased photon penetration, the degradation efficiency somewhat decreased at 30 mg. The degradation rate also progressively dropped as the starting AMX concentration rose from 5 to 20 mg L^−1^, indicating that larger pollutant concentrations lead to more adsorption competition on the catalyst surface, which reduces the number of active sites available for photocatalysis.^57^ The identification of reactive species participating in the process is a crucial aspect of photocatalytic degradation. Using scavengers like benzoquinone (BQ) for superoxide radicals (˙O_2_^−^), isopropanol (IPA) for hydroxyl radicals (˙OH), and ethylenediaminetetraacetic acid (EDTA) for holes (h^+^), radical trapping studies were carried out to comprehend the prevailing degradation process. The findings (Fig. 13(e)) show that the addition of EDTA and IPA considerably reduced the degradation efficiency, suggesting that holes (h^+^) and hydroxyl radicals (˙OH) were the main causes of AMX degradation, whereas the impact of ˙O_2_^−^ was relatively smaller. Photoluminescence (PL) analysis was performed to confirm the improved charge separation in Cu/ZnS@ g-C_3_N_4_ (Fig. 13(f)). The hybrid composite had decreased PL intensity compared to virgin ZnS and g-C_3_N_4_, indicating less charge carrier recombination and better photocatalytic efficiency.^58^ EIS investigations show Cu/ZnS@ g-C_3_N_4_ has much reduced charge transfer resistance, indicating enhanced charge transport capabilities. Based on these observations, the hypothesized photocatalytic degradation pathway is depicted in Fig. 13. When exposed to visible light, Cu/ZnS@ g-C_3_N_4_ absorbs photons and forms electron–hole pairs. Photogenerated electrons pass from the valence band (VB) of g-C_3_N_4_ to the conduction band (CB) of Cu–ZnS, successfully preventing recombination.^59^ The significant photoluminescence quenching observed in steady-state PL measurements (Fig. 13(f)) clearly demonstrates suppressed electron–hole recombination at the heterojunction interface. This is further corroborated by the composite's superior photocatalytic performance, showing 2–3× higher AMX degradation rates compared to individual components, which functionally validates more efficient charge carrier utilization. Electrochemical impedance spectroscopy reveals dramatically reduced charge transfer resistance (from 15.8 to 4.2 Ω cm^2^), indicating improved interfacial electron transport. Mott–Schottky analysis shows the expected flat-band potential shift (−0.96 V *vs.* −1.13 V) characteristic of effective heterojunction formation. Additionally, radical trapping experiments confirm that photogenerated charges are being effectively harnessed for redox reactions rather than recombining, with ˙OH radicals and holes playing dominant roles in the degradation mechanism. The absence of new UV-vis absorption features (Fig. 13(a)), coupled with the dominant role of ˙OH/h^+^ (Fig. 13(e)) and optimal band alignment for ROS generation (Fig. 13(g)), suggests thorough degradation of AMX into small molecules (*e.g.*, CO_2_, NH_4_^+^). Prior studies of analogous systems report >80% TOC removal under comparable conditions.^57,59^ Electrons convert dissolved oxygen to superoxide radicals (˙O_2_^−^), whereas holes oxidize H_2_O to produce hydroxyl radicals (˙OH). These highly reactive species subsequently engage with AMX molecules, converting the antibiotic into smaller, less toxic byproducts.^60^ A schematic energy band diagram showing the charge transfer between Cu–ZnS and g-C_3_N_4_ and photocatalytic mechanisms is presented in Fig. 14.^61^ The exceptional photocatalytic efficacy of Cu/ZnS@g-C_3_N_4_ underscores its promise for wastewater treatment and environmental remediation. This dual-functional material effectively degrades AMX, a prevalent pharmaceutical pollutant, demonstrating its practical use in mitigating water pollution while also functioning as an electrode for energy storage applications. The integration of improved charge transfer, elevated photocatalytic performance, and structural stability renders these hybrid nanostructures attractive candidates for advanced multifunctional materials in renewable energy and environmental sustainability.

## 4. Conclusions

A g-C_3_N_4_/Cu–ZnS hybrid nanostructure was successfully developed in this work, exhibiting dual functionality in photocatalytic and supercapacitor applications. With an exceptional specific capacitance of 275 F g^−1^ at 1 A g^−1^, 70% capacitance retention at 20 A g^−1^, and 92.5% stability after 10 000 cycles, the optimized CuZnS-GCN25 electrode demonstrated its superior charge storage capacity, cycling endurance, and high-rate performance. Its excellent coulombic efficiency (∼98%) and broad potential window (1.6 V) further confirm its suitability for use in hybrid supercapacitors for cutting-edge energy storage devices. Using pseudo-first-order kinetics (*k* = 0.029 min^−1^), CuZnS-GCN25 efficiently broke down 92.4% of amoxicillin (AMX) in 60 min when exposed to visible light for photocatalytic applications. The generation of active species and improved charge separation efficiency were key factors in accelerating the photocatalytic degradation process. It is an efficient catalyst for wastewater treatment applications because of the synergistic interaction between Cu–ZnS and g-C_3_N_4_, which greatly enhanced electron transport, charge carrier mobility, and structural stability. The multifunctional CuZnS-GCN25 composite effectively addresses important issues in both environmental remediation and high-performance energy storage. It has been identified as a potential material for next-generation sustainable energy storage devices and water purification technologies due to its remarkable super capacitive behavior and photocatalytic efficiency.

## Author contributions

J. Khan designed and supervised the project, and performed the experiments. E. A. Alabdullkarem performed the critical analysis of the results obtained from electroanalytical techniques used in the study. Both the authors have equally contributed in writeup of manuscript.

## Conflicts of interest

The authors declare no competing financial interest.

## Data Availability

All data analyzed during the study are included in this article and will be made available upon reasonable request.
